# Supplementary material for: Genome‑wide analysis of the MYB gene family in pumpkin
Source: PeerJ. 2024 Apr 25;12:e17304. doi: 10.7717/peerj.17304 (PMC11056105; doi:10.7717/peerj.17304)
Supplement: Supplemental Information 1 [file peerj-12-17304-s001.docx]

**The Information of RT-qPCR Experiments In This Study**

**1. Experimental design and Samples**

The “TianMiyihao” were used as the experimental material. Seedlings with good growth condition were cultured with Hoagland’s nutrient solution in a greenhouse (28℃, 70–80% humidity, 16 h light/8 h dark). The roots, stems, and leaves of three-leaf seedlings pumpkin were harvested to analyze the expression levels of CmoMYB genes. Meanwhile, uniformly sized three-leaf seedlings were treated with simulated drought treatment (20% PEG6000) and different hormones (100 µM JA, 10 µM ABA, and 100 µM SA). Leaves from each group were obtained after 0, 3, and 6 h of treatment, respectively. The samples were snap-frozen in liquid nitrogen and stored at −80℃. Each sample consisted of three different plant leaves and three experiments were performed.

**2.Nucleic acid extraction and Reverse transcription**

The instruments used during the experiment were frozen centrifuge (Nanodrop 2000, Thermo Fisher Scientific, America), electrophoresis apparatus (DYY-8C, Beijing Liuyi, China), and nucleic acid tester (D3024R, Eppendoef, Germany). Total RNA of each sample was extracted by using Trizol reagent (Takara, Beijing, China), RNase was not available in the operating environment, reagents and consumables. RNA integrity was verified by 1.2% agarose gel electrophoresis and RNA purity and concentration were determined in the nucleic acid tester.

The first strand of cDNA was obtained by the reverse transcribing of 1 µg of RNA according to the manufacturer’s First-Strand cDNA Synthesis Kit (Vazyme, Nanjing, China).

**3.qPCR target information and qPCR oligonucleotides**

The specific primers for the *ComMYB* genes were designed for qRT-PCR analysis using Primer Premier 5 software (Table 1). The Cmoβ-actin gene was used as as the normalization reference gene. The AceQ qPCR SYBR Green Master Mix (Vazyme, Nanjing, China) was used for qRT-PCR.

**Table 1.** The specific primers for the *ComMYB* genes for qRT-PCR analysis.

| **Primer name** | **Sequence (5'to3')** |
| --- | --- |
| CmoMYB99-qF | AGGACCTGTCTCCAACCCAA |
| CmoMYB99-qR | GTTCACATCGCACCCCAATA |
| CmoMYB165-qF | TCTCACGCTATCTCCCTCCG |
| CmoMYB165-qR | CGCCCTTCTTCCAACTCCTT |
| CmoMYB142-qF | CCCAAACATCATCAGGTAAC |
| CmoMYB142-qR | AGCAGACTCATTCAAAACAC |
| CmoMYB154-qF | TTGTTTCAAAATGTCGCCTT |
| CmoMYB154-qR | TTGTTTCTCTCTGTCCTCGC |
| CmoMYB144-qF | CGAACACCGCCCCTTCTCTC |
| CmoMYB144-qR | GGATTTCCGCTGGTCTCTAA |
| CmoMYB116-qF | CTCATTCTCCAACTCCACTC |
| CmoMYB116-qR | CTTGCTTTTGAACTCTCGTC |
| CmoMYB70-qF | GGACAGGGGTGTTGGAGTGA |
| CmoMYB70-qR | TTTGAGGTCGGGTCTAAGGT |
| CmoMYB46-qF | GATTTCAATACCCGCCGCC |
| CmoMYB46-qR | TGCCCCTGTTGTTCTCCTCC |
| CmoMYB64-qF | TGTCATCATTCACCCTGCTG |
| CmoMYB64-qR | TCTCCTTCTTCCTCCCATTT |
| CmoMYB59-qF | AGCCCTCAAGAAGACCAA |
| CmoMYB59-qR | TTCCCGCACCGTAACAGT |
| CmoMYB3-qF | CCGACAGAGAAACGACAT |
| CmoMYB3-qR | GCAAGAAAAGCAAAGACT |
| CmoMYB29-qF | GGAAGAACCGACAATGAGAT |
| CmoMYB29-qR | AGACGACGAGGAAGGAATAC |
| CmoMYB9-qF | AACGGACAATGAAGTAAAGA |
| CmoMYB9-qR | GTAAAAAGATGAACCCAAAA |
| Cmoβ-Actin-qF | GTGCCTGCTATGTATGTTGCC |
| Cmoβ-Actin-qR | GGTCCAAACGGAGAATGGCATG |

**4.qPCR protocol, qPCR validation and Data analysis**

The reaction consisted of 10 µL AceQ qPCR SYBR Green Master Mix (Vazyme, Nanjing, China), 0.2 µmol L-1 upstream and downstream primers, 2.0 µL cDNA, and up to 20 µL with ddH2O. The instrument used was the Thermo Scientific PikoReal Cycler and process was set as follow steps: 95 ℃ for 3 min, followed by 40 cycles of 95 ℃ for 10 s, 60 ℃ for 30 s, and 60 ℃ for 30 s.

The dissolution curve procedure was set as: starting temperature is 60℃; final temperature is 95℃; holding time is 00:01; temperature increment is 0.2℃; the dissolution curves of all primers are single peak map, indicating the specificity of the primer.

The Cq values were determined by the thresholding method, and the Cq values for each sample are shown in the Table 2. Relative expression level of genes was calculated by the 2−∆∆CT method. Three experimental replicates were performed for each sample.

**Table 2.** The Cq values for qRT-PCR of *CmoMYB* genes

| **Name** | **CK** | **PEG3H** | **PEG6H** | **ABA3H** | **ABA6H** | **SA3H** | **SA6H** | **JA3H** | **JA6H** | **Root** | **Steam** | **Leaf** |
| --- | --- | --- | --- | --- | --- | --- | --- | --- | --- | --- | --- | --- |
| *β-Actin* | 19.26 | 20.71 | 21.92 | 20.46 | 20.47 | 21.72 | 20.89 | 23.4 | 20.63 | 33.27 | 25.23 | 19.26 |
|  | 19.23 | 20.48 | 22.01 | 20.91 | 20.64 | 21.7 | 20.76 | 23.99 | 20.72 | 32.52 | 24.95 | 19.23 |
|  | 19.27 | 20.61 | 22.08 | 20.66 | 20.68 | 21.7 | 20.91 | 23.88 | 21.07 | 32.72 | 25.12 | 19.27 |
| *ComMYB99* | 35.8 | 36.08 | 32.97 | 37.3 | 32.86 | 34.9 | 34.59 | 34.6 | 33.79 | 34.4 | 34.59 | 35.8 |
|  | 36.52 | 35.73 | 33 | 36.52 | 33.17 | 34.48 | 34.3 | 34.81 | 33.68 | 35.94 | 34.98 | 36.52 |
|  | 35.38 | 35.8 | 33.62 | 37 | 33.24 | 34.6 | 34.76 | 34.7 | 34.03 | 35 | 33.36 | 35.38 |
| *β-Actin* | 20.85 | 21.99 | 23.78 | 22.11 | 21.69 | 21.48 | 20.54 | 23.75 | 20.14 | 22.07 | 22.49 | 20.85 |
|  | 20.76 | 22.2 | 23.73 | 21.74 | 21.84 | 21.77 | 20.37 | 24.19 | 20.3 | 21.72 | 22.12 | 20.76 |
|  | 20.64 | 21.97 | 23.43 | 22.02 | 21.9 | 21.45 | 20.15 | 24 | 20.25 | 22.12 | 22.04 | 20.64 |
| *ComMYB165* | 24.15 | 26.05 | 26.52 | 25.85 | 24.43 | 25.43 | 26.39 | 26.06 | 23.17 | 27.34 | 27.44 | 24.15 |
|  | 24.02 | 25.95 | 25.94 | 25 | 24.69 | 25.54 | 26.15 | 26.51 | 23.18 | 27.21 | 27.5 | 24.02 |
|  | 23.86 | 26.02 | 26.07 | 25.97 | 24.71 | 25.31 | 25.91 | 26.17 | 23.08 | 27.15 | 27.29 | 23.86 |
| *β-Actin* | 20.85 | 21.99 | 23.78 | 22.11 | 21.69 | 21.48 | 20.54 | 23.75 | 20.14 | 22.07 | 22.49 | 20.85 |
|  | 20.76 | 22.2 | 23.73 | 21.74 | 21.84 | 21.77 | 20.37 | 24.19 | 20.3 | 21.72 | 22.12 | 20.76 |
|  | 20.64 | 21.97 | 23.43 | 22.02 | 21.9 | 21.45 | 20.15 | 24 | 20.25 | 22.12 | 22.04 | 20.64 |
| *ComMYB142* | 26.39 | 28.07 | 27.17 | 27.59 | 26.85 | 24.78 | 27.55 | 28.34 | 25.99 | 27.8 | 28.52 | 26.39 |
|  | 26.39 | 28.22 | 27.18 | 27 | 26.79 | 25.07 | 27.86 | 28.51 | 26.02 | 27.73 | 28.08 | 26.39 |
|  | 26.31 | 28.46 | 27.11 | 27.23 | 26.9 | 25 | 27.57 | 28.57 | 25.88 | 27.26 | 28.63 | 26.31 |
| *β-Actin* | 20.85 | 21.99 | 23.78 | 22.11 | 21.69 | 21.48 | 20.54 | 23.75 | 20.14 | 22.07 | 22.49 | 20.85 |
|  | 20.76 | 22.2 | 23.73 | 21.74 | 21.84 | 21.77 | 20.37 | 24.19 | 20.3 | 21.72 | 22.12 | 20.76 |
|  | 20.64 | 21.97 | 23.43 | 22.02 | 21.9 | 21.45 | 20.15 | 24 | 20.25 | 22.12 | 22.04 | 20.64 |
| *ComMYB154* | 31.87 | 33.62 | 34.87 | 33.88 | 33.87 | 31.08 | 33.99 | 35.42 | 30.66 | 29.82 | 34.67 | 31.87 |
|  | 31.65 | 34 | 34.76 | 33.55 | 34.09 | 31.21 | 33.21 | 35.29 | 30.68 | 29.84 | 34.51 | 31.65 |
|  | 31.58 | 33.49 | 34.51 | 33.29 | 33.45 | 31 | 33.97 | 35.93 | 30.95 | 29.84 | 34.17 | 31.58 |
| *β-Actin* | 20.85 | 21.99 | 23.78 | 22.11 | 21.69 | 21.48 | 20.54 | 23.75 | 20.14 | 22.07 | 22.49 | 20.85 |
|  | 20.76 | 22.2 | 23.73 | 21.74 | 21.84 | 21.77 | 20.37 | 24.19 | 20.3 | 21.72 | 22.12 | 20.76 |
|  | 20.64 | 21.97 | 23.43 | 22.02 | 21.9 | 21.45 | 20.15 | 24 | 20.25 | 22.12 | 22.04 | 20.64 |
| *ComMYB144* | 28.51 | 27.63 | 27.98 | 23.97 | 25.96 | 28.05 | 25.82 | 27.51 | 23.41 | 26.17 | 27.58 | 28.51 |
|  | 28.18 | 27.69 | 27.91 | 23.37 | 25.58 | 28.04 | 25.49 | 27.61 | 23.59 | 26.13 | 27.76 | 28.18 |
|  | 28.74 | 27.49 | 27.34 | 23.63 | 25.51 | 27.89 | 25.24 | 27.55 | 23.35 | 26.18 | 28.1 | 28.74 |
| *β-Actin* | 20.85 | 21.99 | 23.78 | 22.11 | 21.69 | 21.48 | 20.54 | 23.75 | 20.14 | 22.07 | 22.49 | 20.85 |
|  | 20.76 | 22.2 | 23.73 | 21.74 | 21.84 | 21.77 | 20.37 | 24.19 | 20.3 | 21.72 | 22.12 | 20.76 |
|  | 20.64 | 21.97 | 23.43 | 22.02 | 21.9 | 21.45 | 20.15 | 24 | 20.25 | 22.12 | 22.04 | 20.64 |
| *ComMYB116* | 34.39 | 35.06 | 35 | 34.31 | 34.46 | 32.19 | 31.59 | 33.25 | 30.58 | 27.31 | 30.43 | 34.39 |
|  | 33.94 | 35.2 | 34.46 | 34.4 | 34.41 | 32.62 | 32.3 | 33.77 | 30.76 | 27.48 | 30.86 | 33.94 |
|  | 34.4 | 35.48 | 34.4 | 34.42 | 34.57 | 32.51 | 32.19 | 33.41 | 30.95 | 27.42 | 30.55 | 34.4 |
| *β-Actin* | 20.85 | 21.99 | 23.78 | 22.11 | 21.69 | 21.48 | 20.54 | 23.75 | 20.14 | 22.07 | 22.49 | 20.85 |
|  | 20.76 | 22.2 | 23.73 | 21.74 | 21.84 | 21.77 | 20.37 | 24.19 | 20.3 | 21.72 | 22.12 | 20.76 |
|  | 20.64 | 21.97 | 23.43 | 22.02 | 21.9 | 21.45 | 20.15 | 24 | 20.25 | 22.12 | 22.04 | 20.64 |
| *ComMYB70* | 31.83 | 34.35 | 31.08 | 32.05 | 30.51 | 29.52 | 32 | 33.44 | 28.43 | 30.85 | 32.51 | 31.83 |
|  | 31.74 | 33.93 | 31.19 | 30.97 | 30.45 | 29.61 | 31.56 | 33.82 | 28.58 | 30.56 | 32.21 | 31.74 |
|  | 32.03 | 33.91 | 31.04 | 31.22 | 30.52 | 29.57 | 32.09 | 33.49 | 28.36 | 31.04 | 32.37 | 32.03 |
| *β-Actin* | 20.85 | 21.99 | 23.78 | 22.11 | 21.69 | 21.48 | 20.54 | 23.75 | 20.14 | 22.07 | 22.49 | 20.85 |
|  | 20.76 | 22.2 | 23.73 | 21.74 | 21.84 | 21.77 | 20.37 | 24.19 | 20.3 | 21.72 | 22.12 | 20.76 |
|  | 20.64 | 21.97 | 23.43 | 22.02 | 21.9 | 21.45 | 20.15 | 24 | 20.25 | 22.12 | 22.04 | 20.64 |
| *ComMYB46* | 30.79 | 32.58 | 31.63 | 30.41 | 30.63 | 29.01 | 31.13 | 32.26 | 27.67 | 29.11 | 30.21 | 30.79 |
|  | 30.92 | 33.65 | 31.39 | 30.02 | 30.96 | 29.23 | 30.99 | 32.19 | 27.9 | 29.25 | 30.73 | 30.92 |
|  | 30.55 | 33.13 | 31.01 | 30.36 | 31.04 | 28.83 | 30.53 | 32.1 | 27.8 | 29.21 | 30.75 | 30.55 |
| *β-Actin* | 20.85 | 21.99 | 23.78 | 22.11 | 21.69 | 21.48 | 20.54 | 23.75 | 20.14 | 22.07 | 22.49 | 20.85 |
|  | 20.76 | 22.2 | 23.73 | 21.74 | 21.84 | 21.77 | 20.37 | 24.19 | 20.3 | 21.72 | 22.12 | 20.76 |
|  | 20.64 | 21.97 | 23.43 | 22.02 | 21.9 | 21.45 | 20.15 | 24 | 20.25 | 22.12 | 22.04 | 20.64 |
| *ComMYB64* | 34.54 | 34.68 | 37.33 | 32 | 35.42 | 30.62 | 35.63 | 36.53 | 31.78 | 33.45 | 34.42 | 33.38 |
|  | 34.48 | 34.86 | 37.27 | 31.66 | 35.64 | 30.89 | 35.56 | 36.31 | 31.94 | 33.42 | 34.01 | 34.48 |
|  | 34 | 34.53 | 36.55 | 31.65 | 36.03 | 30.68 | 34.59 | 36.56 | 32.05 | 34.71 | 34.05 | 34.54 |
| *β-Actin* | 20.85 | 21.99 | 23.78 | 22.11 | 21.69 | 21.48 | 20.54 | 23.75 | 20.14 | 22.07 | 22.49 | 20.85 |
|  | 20.76 | 22.2 | 23.73 | 21.74 | 21.84 | 21.77 | 20.37 | 24 | 20.3 | 21.72 | 22.12 | 20.76 |
|  | 20.64 | 21.97 | 23.43 | 22.02 | 21.9 | 21.45 | 20.15 | 24.19 | 20.25 | 22.12 | 22.04 | 20.64 |
| *ComMYB59* | 33.6 | 36.91 | 34.87 | 32.3 | 37.28 | 32.82 | 36.09 | 36.92 | 33.46 | 35.31 | 35.02 | 33.33 |
|  | 33.06 | 37.16 | 34.37 | 32.11 | 35.89 | 32.78 | 37.63 | 36.59 | 34.18 | 35.46 | 34.45 | 33.06 |
|  | 34.16 | 37.8 | 34.13 | 32.74 | 35.32 | 32.2 | 37.52 | 37.2 | 33.16 | 35.33 | 34.8 | 33.16 |
| *β-Actin* | 20.85 | 20.78 | 23.78 | 22.11 | 21.69 | 21.48 | 20.54 | 23.75 | 20.14 | 22.07 | 22.49 | 20.85 |
|  | 20.76 | 20.51 | 23.73 | 21.74 | 21.84 | 21.77 | 20.37 | 24.19 | 20.3 | 21.72 | 22.12 | 20.76 |
|  | 20.64 | 20.92 | 23.43 | 22.02 | 21.9 | 21.45 | 20.15 | 24 | 20.25 | 22.12 | 22.04 | 20.64 |
| *ComMYB3* | 28.05 | 25.45 | 27.71 | 26.3 | 27.16 | 26.55 | 27.19 | 31.23 | 25.18 | 25.44 | 30.47 | 28.05 |
|  | 28.02 | 25.44 | 27.93 | 26.1 | 27.08 | 26.97 | 27.36 | 31.22 | 25.08 | 25.24 | 30.19 | 28.02 |
|  | 28.06 | 25.55 | 27.68 | 26.22 | 27.28 | 26.86 | 27.89 | 31.35 | 25.12 | 25.55 | 30.5 | 28.06 |
| *β-Actin* | 26.17 | 20.91 | 22.09 | 20.23 | 21.05 | 20.43 | 20.04 | 23.22 | 21.19 | 23.22 | 23.22 | 26.17 |
|  | 26.15 | 21.03 | 22.39 | 20.05 | 21.01 | 20.29 | 20.44 | 23.66 | 21.28 | 23.66 | 23.66 | 26.15 |
|  | 26.18 | 21.51 | 22.55 | 20.41 | 20.95 | 20.42 | 20.14 | 23.14 | 21.23 | 23.14 | 23.14 | 26.18 |
| *ComMYB29* | 31.66 | 26.4 | 27.66 | 24.18 | 26.3 | 26.55 | 27.45 | 36.83 | 27.05 | 36.83 | 35.73 | 31.66 |
|  | 31.34 | 26.33 | 28.05 | 24.27 | 26.32 | 26.6 | 27.03 | 35.07 | 26.5 | 35.07 | 35.58 | 31.34 |
|  | 31.54 | 26.9 | 28.52 | 24.36 | 26.37 | 26.13 | 27.92 | 35.11 | 27.05 | 35.11 | 36.97 | 31.54 |
| *β-Actin* | 20.85 | 20.78 | 23.78 | 22.11 | 21.69 | 21.48 | 20.54 | 23.75 | 20.14 | 22.07 | 22.49 | 20.85 |
|  | 20.76 | 20.51 | 23.73 | 21.74 | 21.84 | 21.77 | 20.37 | 24.19 | 20.3 | 21.72 | 22.12 | 20.76 |
|  | 20.64 | 20.92 | 23.43 | 22.02 | 21.9 | 21.45 | 20.15 | 24 | 20.25 | 22.12 | 22.04 | 20.64 |
| *ComMYB9* | 28.09 | 30.19 | 28.41 | 27.05 | 25.85 | 25.07 | 27.76 | 31.39 | 28.57 | 27.33 | 31.03 | 28.09 |
|  | 28.75 | 29.81 | 28.56 | 26.9 | 26.26 | 25.42 | 28.23 | 31.4 | 28.65 | 27.23 | 29.15 | 28.75 |
|  | 29.44 | 31.39 | 28.3 | 27.44 | 26.51 | 24.95 | 28.05 | 31.27 | 29.17 | 27.09 | 29.47 | 29.44 |
