# Supplementary material for: Genome‑wide analysis of the MYB gene family in pumpkin
Source: PeerJ. 2024 Apr 25;12:e17304. doi: 10.7717/peerj.17304 (PMC11056105; doi:10.7717/peerj.17304)
Supplement: Supplemental Information 3 [file peerj-12-17304-s003.docx]

**Table S1:** The information of the identified *CmoMYB* genes.

| **Gene name** | **Gene ID** | **MYB**  **domain** | **PI** | **MW (kDa)** | **Chromosome Location** | **CDS**  **Length (bp)** | **Number of**  **Amino Acids (aa)** | **GRAVY** | **Subcellular Localization** |
| --- | --- | --- | --- | --- | --- | --- | --- | --- | --- |
| *CmoMYB1*  *CmoMYB2*  *CmoMYB3*  *CmoMYB4*  *CmoMYB5*  *CmoMYB6*  *CmoMYB7*  *CmoMYB8*  *CmoMYB9*  *CmoMYB10*  *CmoMYB11*  *CmoMYB12*  *CmoMYB13*  *CmoMYB14*  *CmoMYB15*  *CmoMYB16*  *CmoMYB17*  *CmoMYB18*  *CmoMYB19*  *CmoMYB20*  *CmoMYB21*  *CmoMYB22*  *CmoMYB23*  *CmoMYB24*  *CmoMYB25*  *CmoMYB26*  *CmoMYB27*  *CmoMYB28*  *CmoMYB29*  *CmoMYB30*  *CmoMYB31*  *CmoMYB32*  *CmoMYB33*  *CmoMYB34*  *CmoMYB35*  *CmoMYB36*  *CmoMYB37*  *CmoMYB38*  *CmoMYB39*  *CmoMYB40*  *CmoMYB41*  *CmoMYB42*  *CmoMYB43*  *CmoMYB44*  *CmoMYB45*  *CmoMYB46*  *CmoMYB47*  *CmoMYB48*  *CmoMYB49*  *CmoMYB50*  *CmoMYB51*  *CmoMYB52*  *CmoMYB53*  *CmoMYB54*  *CmoMYB55*  *CmoMYB56*  *CmoMYB57*  *CmoMYB58*  *CmoMYB59*  *CmoMYB60*  *CmoMYB61*  *CmoMYB62*  *CmoMYB63*  *CmoMYB64*  *CmoMYB65*  *CmoMYB66*  *CmoMYB67*  *CmoMYB68*  *CmoMYB69*  *CmoMYB70*  *CmoMYB71*  *CmoMYB72*  *CmoMYB73*  *CmoMYB74*  *CmoMYB75*  *CmoMYB76*  *CmoMYB77*  *CmoMYB78*  *CmoMYB79*  *CmoMYB80*  *CmoMYB81*  *CmoMYB82*  *CmoMYB83*  *CmoMYB84*  *CmoMYB85*  *CmoMYB86*  *CmoMYB87*  *CmoMYB88*  *CmoMYB89*  *CmoMYB90*  *CmoMYB91*  *CmoMYB92*  *CmoMYB93*  *CmoMYB94*  *CmoMYB95*  *CmoMYB96*  *CmoMYB97*  *CmoMYB98*  *CmoMYB99*  *CmoMYB100*  *CmoMYB101*  *CmoMYB102*  *CmoMYB103*  *CmoMYB104*  *CmoMYB105*  *CmoMYB106*  *CmoMYB107*  *CmoMYB108*  *CmoMYB109*  *CmoMYB110*  *CmoMYB111*  *CmoMYB112*  *CmoMYB113*  *CmoMYB114*  *CmoMYB115*  *CmoMYB116*  *CmoMYB117*  *CmoMYB118*  *CmoMYB119*  *CmoMYB120*  *CmoMYB121*  *CmoMYB122*  *CmoMYB123*  *CmoMYB124*  *CmoMYB125*  *CmoMYB126*  *CmoMYB127*  *CmoMYB128*  *CmoMYB129*  *CmoMYB130*  *CmoMYB131*  *CmoMYB132*  *CmoMYB133*  *CmoMYB134*  *CmoMYB135*  *CmoMYB136*  *CmoMYB137*  *CmoMYB138*  *CmoMYB139*  *CmoMYB140*  *CmoMYB141*  *CmoMYB142*  *CmoMYB143*  *CmoMYB144*  *CmoMYB145*  *CmoMYB146*  *CmoMYB147*  *CmoMYB148*  *CmoMYB149*  *CmoMYB150*  *CmoMYB151*  *CmoMYB152*  *CmoMYB153*  *CmoMYB154*  *CmoMYB155*  *CmoMYB156*  *CmoMYB157*  *CmoMYB158*  *CmoMYB159*  *CmoMYB160*  *CmoMYB161*  *CmoMYB162*  *CmoMYB163*  *CmoMYB164*  *CmoMYB165*  *CmoMYB166*  *CmoMYB167*  *CmoMYB168*  *CmoMYB169*  *CmoMYB170*  *CmoMYB171*  *CmoMYB172*  *CmoMYB173*  *CmoMYB174*  *CmoMYB175* | CmoCh20G007520.1  CmoCh02G002520.1  CmoCh11G017310.1  CmoCh09G013230.1  CmoCh01G008740.1  CmoCh20G010400.1  CmoCh19G007410.1  CmoCh03G005180.1  CmoCh07G002730.1  CmoCh19G009880.1  CmoCh13G007030.1  CmoCh18G003180.1  CmoCh15G002700.1  CmoCh04G029050.1  CmoCh03G008480.1  CmoCh14G020890.1  CmoCh06G011140.1  CmoCh05G010620.1  CmoCh10G012670.1  CmoCh19G002380.1  CmoCh15G012170.1  CmoCh11G000750.1  CmoCh02G016690.1  CmoCh12G008720.1  CmoCh18G001470.1  CmoCh15G013710.1  CmoCh14G008430.1  CmoCh14G005780.1  CmoCh01G003920.1  CmoCh14G009510.1  CmoCh14G006260.1  CmoCh20G009160.1  CmoCh15G014530.1  CmoCh02G015660.1  CmoCh15G007700.1  CmoCh14G016310.1  CmoCh14G007130.1  CmoCh06G015520.1  CmoCh11G012520.1  CmoCh01G003060.1  CmoCh10G001040.1  CmoCh02G000100.1  CmoCh14G012830.1  CmoCh20G003020.1  CmoCh08G010590.1  CmoCh06G007010.1  CmoCh19G001010.1  CmoCh13G008960.1  CmoCh17G001630.1  CmoCh06G013300.1  CmoCh14G004790.1  CmoCh03G002010.1  CmoCh12G010150.1  CmoCh04G028930.1  CmoCh10G001800.1  CmoCh07G000100.1  CmoCh06G000480.1  CmoCh14G018510.1  CmoCh08G002890.1  CmoCh14G006020.1  CmoCh01G005450.1  CmoCh13G011860.1  CmoCh04G023920.1  CmoCh03G009230.1  CmoCh02G001360.1  CmoCh01G000450.1  CmoCh09G003180.1  CmoCh01G008230.1  CmoCh07G006280.1  CmoCh01G002350.1  CmoCh02G014200.1  CmoCh11G010480.1  CmoCh14G010210.1  CmoCh02G012850.1  CmoCh19G009840.1  CmoCh11G001610.1  CmoCh02G014610.1  CmoCh14G012670.1  CmoCh17G009280.1  CmoCh20G000840.1  CmoCh05G001200.1  CmoCh01G018180.1  CmoCh12G005570.1  CmoCh15G003560.1  CmoCh04G029420.1  CmoCh11G004090.1  CmoCh14G006720.1  CmoCh12G012230.1  CmoCh04G026110.1  CmoCh05G012980.1  CmoCh11G018940.1  CmoCh02G010780.1  CmoCh06G003880.1  CmoCh19G009420.1  CmoCh04G028290.1  CmoCh15G007040.1  CmoCh11G016850.1  CmoCh05G008320.1  CmoCh04G010390.1  CmoCh11G006820.1  CmoCh11G019240.1  CmoCh13G002400.1  CmoCh17G003460.1  CmoCh11G019280.1  CmoCh08G012310.1  CmoCh17G009910.1  CmoCh06G015690.1  CmoCh01G005960.1  CmoCh13G010630.1  CmoCh06G003950.1  CmoCh08G004800.1  CmoCh11G002830.1  CmoCh14G018970.1  CmoCh01G005970.1  CmoCh15G005500.1  CmoCh01G002810.1  CmoCh10G004430.1  CmoCh02G017900.1  CmoCh14G016090.1  CmoCh16G010140.1  CmoCh13G001580.1  CmoCh04G003730.1  CmoCh16G002280.1  CmoCh17G005000.1  CmoCh08G010240.1  CmoCh01G018660.1  CmoCh04G001420.1  CmoCh20G009820.1  CmoCh05G001630.1  CmoCh02G008330.1  CmoCh12G005160.1  CmoCh16G003650.1  CmoCh11G000920.1  CmoCh15G003580.1  CmoCh02G005070.1  CmoCh04G028270.1  CmoCh07G010800.1  CmoCh14G001400.1  CmoCh07G006920.1  CmoCh04G005040.1  CmoCh03G003930.1  CmoCh06G002970.1  CmoCh12G004570.1  CmoCh01G019250.1  CmoCh01G016480.1  CmoCh05G006280.1  CmoCh12G002160.1  CmoCh09G002010.1  CmoCh13G012040.1  CmoCh09G005440.1  CmoCh10G001270.1  CmoCh13G000650.1  CmoCh01G005140.1  CmoCh06G000610.1  CmoCh18G011620.1  CmoCh04G017460.1  CmoCh14G004630.1  CmoCh04G025320.1  CmoCh20G005930.1  CmoCh14G008130.1  CmoCh09G010810.1  CmoCh14G009690.1  CmoCh01G007870.1  CmoCh20G004650.1  CmoCh01G004180.1  CmoCh20G010680.1  CmoCh02G006370.1  CmoCh04G017190.1  CmoCh18G011890.1  CmoCh15G006410.1  CmoCh15G011000.1  CmoCh02G011270.1  CmoCh10G003440.1  CmoCh11G009940.1  CmoCh10G010750.1 | R2R3  R2R3  R2R3  R2R3  R2R3  R2R3  1R  R2R3  R2R3  R2R3  R2R3  R2R3  R2R3  R2R3  R2R3  R2R3  R2R3  R2R3  R2R3  R2R3  R2R3  R2R3  R2R3  R2R3  R2R3  R2R3  R2R3  R2R3  R2R3  R2R3  R2R3  R2R3  R2R3  R2R3  R2R3  R2R3  R2R3  R2R3  R2R3  R2R3  R2R3  R2R3  R2R3  R2R3  R2R3  1R  R2R3  R2R3  R2R3  R2R3  R2R3  R2R3  R2R3  R2R3  R2R3  R2R3  R2R3  R2R3  R2R3  R2R3  R2R3  R2R3  R2R3  R2R3  R2R3  R2R3  R2R3  R2R3  R2R3  R2R3  R2R3  R2R3  R2R3  R2R3  R2R3  R2R3  R2R3  R2R3  R2R3  R2R3  R2R3  R2R3  R2R3  R2R3  R2R3  R2R3  R2R3  R2R3  R2R3  R2R3  R2R3  R2R3  R2R3  R2R3  R2R3  R2R3  R2R3  R2R3  R2R3  R2R3  R2R3  1R  1R  1R  1R  R2R3  R2R3  R2R3  R2R3  R2R3  R2R3  R2R3  R2R3  R2R3  3R  R2R3  R2R3  R2R3  R2R3  R2R3  R2R3  R2R3  R2R3  R2R3  1R  R2R3  R2R3  R2R3  R2R3  R2R3  R2R3  R2R3  1R  1R  1R  1R  1R  R2R3  1R  R2R3  1R  R2R3  R2R3  R2R3  3R  3R  3R  R2R3  R2R3  R2R3  1R  R2R3  R2R3  1R  R2R3  R2R3  R2R3  1R  R2R3  R2R3  R2R3  1R  R2R3  1R  R2R3  R2R3  R2R3  R2R3  R2R3  R2R3  1R  1R  4R  R2R3  1R | 8.28  8.59  9.03  8.67  8.79  9.15  8.94  5.82  5.67  5.34  6.4  6.26  5.98  6.72  6.05  5.74  5.2  8.95  6.86  6.67  6.01  6.67  7.06  8.41  6.79  5.67  6.31  9.6  5.72  4.61  8.62  6.09  6.52  6.08  6.4  6.95  8.41  6.35  6.2  8.17  6.28  5.1  7.05  4.92  8.63  5.76  6.98  6.07  6.03  9.01  5.69  7.59  8.33  8.07  6.02  6.31  6.92  8.99  6.26  5.75  7.19  8.05  5.96  9.85  6.32  7.7  8.04  8.66  6.6  5.22  8.7  6.01  5.46  6.08  6.16  9.22  6.08  7.15  5.59  8.43  8.52  8.32  9.09  5.3  6.15  6.42  6.3  7.05  5.7  6.32  8.73  8.28  8.96  6.75  5.67  5.4  9.16  5.88  6.66  5.59  8.9  8.93  9.33  9.59  10.04  5.9  5.19  8.58  8.76  8.97  5.6  6.6  8.69  6.54  5.66  7.13  6.76  6.19  6.12  6.51  6.08  6.56  5.3  5.44  9.35  8.18  6.67  9.44  6.22  9.2  8.9  9  5.93  9.05  5.32  6.49  5.14  8.5  8.7  5.63  5.36  9.13  10.26  9.27  5.7  5.37  5.13  9.21  9.54  5.3  6.76  5.63  10.61  9.46  8.85  6.75  9.88  9.07  5.18  9.31  10.24  5.56  11.04  10.51  9.49  10.35  10.11  7.24  7.61  9.87  9.39  6.91  6.05  6.39  5.12 | 28.69864  27.2691  27.61151  30.79356  31.30412  24.74196  24.89825  39.15353  41.96652  37.60017  34.87132  34.51894  38.12535  54.90324  38.72077  33.77381  35.54665  26.68118  41.51335  34.68689  26.3226  38.74609  32.11948  29.75074  36.79645  28.33261  27.62214  28.98147  28.30392  35.25993  31.02307  34.44164  26.4497  30.57605  30.93695  30.66265  30.02318  30.59712  46.19157  96.93764  41.25395  36.88487  38.31651  38.05423  28.32269  41.4515  27.40909  41.86896  29.0835  34.41696  30.85762  32.91578  27.04865  32.28141  44.03162  36.74929  31.7418  30.34535  30.39157  34.30527  30.66527  90.27672  36.39635  22.56593  27.68095  22.63047  32.75991  31.10891  35.70717  35.543  26.45201  35.04793  36.53396  32.57742  28.97452  28.37197  36.77807  50.1777  35.72506  41.69993  37.90962  65.28847  36.08654  24.31818  23.60874  36.53391  23.38937  27.71697  61.02562  29.57098  44.08682  25.6086  38.03547  46.99586  24.25714  29.86523  23.75097  24.2333  55.37364  30.16782  30.60906  34.39172  11.02257  10.12262  11.08186  29.97382  33.36886  89.74148  54.7508  27.65715  29.42593  32.07955  31.97814  25.09232  102.56289  29.13078  40.45368  73.7586  32.54112  34.56912  27.66731  35.87954  35.89107  34.60589  11.27196  31.76872  30.67528  31.40657  76.66806  51.67555  29.14183  25.9924  51.71455  49.39865  32.02675  49.29131  40.54721  60.4843  33.20315  19.21139  40.94561  61.45383  28.393  24.63737  105.35635  105.17986  111.82866  25.35811  26.96823  113.80503  28.68711  47.91966  19.32192  33.52849  100.69076  36.11744  29.30931  33.3623  53.99038  42.97695  27.55613  20.59988  15.74711  12.49564  43.87632  24.81699  24.84195  72.38888  51.30029  36.46076  9.83328  9.80311  112.9172  84.93156  114.86167 | Chr20: 3778404...3779972(+)  Chr02: 1190454...1200490(-)  Chr16: 1397496...12247767(+)  Chr34: 3985446...11980609(-)  Chr52: 6573396...4719053(+)  Chr70: 9161346...9498130(-)  Chr88: 11749296...7617597(+)  Chr106:14337246...5371084(-)  Chr124:16925196...1342322(+)  Chr142:19513146...8846674(-)  Chr160:22101096...7160400(+)  Chr178:24689046...2048644(-)  Chr196:27276996...1297235(+)  Chr214:29864946...20652055(-)  Chr232:32452896...6708836(+)  Chr250:35040846...15180878(-)  Chr268:37628796...8582027(+)  Chr286:40216746...8560563(-)  Chr304:42804696...13057049(+)  Chr322:45392646...1683546(-)  Chr340:47980596...8417325(+)  Chr358:50568546...352716(-)  Chr376:53156496...9581813(+)  Chr394:55744446...7943058(-)  Chr412:58332396...1040897(+)  Chr430:60920346...9345305(-)  Chr448:63508296...4442363(+)  Chr466:66096246...2951634(-)  Chr484:68684196...1939684(+)  Chr502:71272146...5077338(-)  Chr520:73860096...3145391(+)  Chr538:76448046...4702366(-)  Chr556:79035996...9856363(+)  Chr574:81623946...9048980(-)  Chr592:84211896...3785735(+)  Chr610:86799846...12962161(-)  Chr628:89387796...3662411(+)  Chr646:91975746...10919845(-)  Chr664:94563696...7935818(+)  Chr682:97151646...1465842(-)  Chr700:99739596...453729(+)  Chr718:102327546...82038(-)  Chr736:104915496...10752642(+)  Chr754:107503446...1477857(-)  Chr772:110091396...6836086(+)  Chr790:112679346...3556106(-)  Chr808:115267296...579067(+)  Chr826:117855246...8019579(-)  Chr844:120443196...945206(+)  Chr862:123031146...9911952(-)  Chr880:125619096...2370465(+)  Chr898:128207046...3315215(-)  Chr916:130794996...9490603(+)  Chr934:133382946...20598545(-)  Chr952:135970896...812167(+)  Chr970:138558846...75413(-)  Chr988:141146796...307518(+)  Chr1006:143734746...13982380(-)  Chr1024:146322696...1787639(+)  Chr1042:148910646...3050009(-)  Chr1060:151498596...2701068(+)  Chr1078:154086546...9416297(-)  Chr1096:156674496...17831568(+)  Chr1114:159262446...7047400(-)  Chr1132:161850396...684216(+)  Chr1150:164438346...142199(-)  Chr1168:167026296...1382069(+)  Chr1186:169614246...4409034(-)  Chr1204:172202196...2841538(+)  Chr1222:174790146...1061582(-)  Chr1240:177378096...8400183(+)  Chr1258:179966046...5837904(-)  Chr1276:182553996...5562873(+)  Chr1294:185141946...7667809 (-)  Chr1312:187729896...8835258(+)  Chr1330:190317846...809565(-)  Chr1348:192905796...8595392(+)  Chr1366:195493746...10631344(-)  Chr1384:198081696...8273525(+)  Chr1402:200669646...438929(-)  Chr1420:203257596...514184(+)  Chr1438:205845546...13337723(-)  Chr1456:208433496...3431048(+)  Chr1474:211021446...1670819(-)  Chr1492:213609396...20840133(+)  Chr1510:216197346...2017012(-)  Chr1528:218785296...3419449(+)  Chr1546:221373246...10916265(-)  Chr1564:223961196...19045480(+)  Chr1582:226549146...10079521(-)  Chr1600:229137096...13163957(+)  Chr1618:231725046...6570042(-)  Chr1636:234312996...1896786(+)  Chr1654:236900946...8654672(-)  Chr1672:239488896...20252139(+)  Chr1690:242076846...3426846(-)  Chr1708:244664796...11948048(+)  Chr1726:247252746...5074123(-)  Chr1744:249840696...5184444(+)  Chr1762:252428646...3275052(-)  Chr1780:255016596...13343765(+)  Chr1798:257604546...1941426(-)  Chr1816:260192496...2091372(+)  Chr1834:262780446...13358412(-)  Chr1852:265368396...7798610(+)  Chr1870:267956346...8565909(-)  Chr1888:270544296...11060541(+)  Chr1906:273132246...3028819(-)  Chr1924:275720196...8837139(+)  Chr1942:278308146...1929379(-)  Chr1960:280896096...2966721(+)  Chr1978:283484046...1375700(-)  Chr1996:286071996...14164946(+)  Chr2014:288659946...3025159(-)  Chr2032:291247896...2638282(+)  Chr2050:293835846...1332856(-)  Chr2068:296423796...2007834(+)  Chr2086:299011746...10188627(-)  Chr2104:301599696...12784062(+)  Chr2122:304187646...6996329(-)  Chr2140:306775596...1010945(+)  Chr2158:309363546...1818597(-)  Chr2176:311951496...1007875(+)  Chr2194:314539446...4222097(-)  Chr2212:317127396...6673985(+)  Chr2230:319715346...13542671(-)  Chr2248:322303296...735966(+)  Chr2266:324891246...5511833(-)  Chr2284:327479196...702772(+)  Chr2302:330067146...5071099(-)  Chr2320:332655096...3171734(+)  Chr2338:335243046...1672821(-)  Chr2356:337830996...452379(+)  Chr2374:340418946...1684817(-)  Chr2392:343006896...2786572(+)  Chr2410:345594846...20244220(-)  Chr2428:348182796...5613336(+)  Chr2446:350770746...620533(-)  Chr2464:353358696...3129232(+)  Chr2482:355946646...2512575(-)  Chr2500:358534596...4798757(+)  Chr2518:361122546...1484673(-)  Chr2536:363710496...2799560(+)  Chr2554:366298446...13777073(-)  Chr2572:368886396...12501271(+)  Chr2590:371474346...3166061(-)  Chr2608:374062296...1428370(+)  Chr2626:376650246...917645(-)  Chr2644:379238196...9461901(+)  Chr2662:381826146...2596047(-)  Chr2680:384414096...582704(+)  Chr2698:387002046...402901(-)  Chr2716:389589996...2471014(+)  Chr2734:392177946...388065(-)  Chr2752:394765896...11814214(+)  Chr2770:397353846...8819112(-)  Chr2788:399941796...2234627(+)  Chr2806:402529746...18606095(-)  Chr2824:405117696...2928897(+)  Chr2842:407705646...4194953(-)  Chr2860:410293596...5878844(+)  Chr2878:412881546...5172834(-)  Chr2896:415469496...4136971(+)  Chr2914:418057446...2156170(-)  Chr2932:420645396...2048710(+)  Chr2950:423233346...9896484(-)  Chr2968:425821296...3967135(+)  Chr2986:428409246...8713018(-)  Chr3004:430997196...11947227(+)  Chr3022:433585146...3116263(-)  Chr3040:436173096...7382314(+)  Chr3058:438761046...6840854(-)  Chr3076:441348996...1561143(+)  Chr3094:443936946...5327518(-)  Chr3112:446524896...5808034(+) | 759  726  744  843  849  666  672  1035  1119  1011  921  918  1035  1506  1050  900  948  702  1116  924  693  1032  861  783  984  771  735  792  756  954  843  921  711  804  825  810  789  795  1230  2622  1095  969  1041  993  753  1125  744  1113  777  924  834  879  735  864  1158  978  861  798  780  918  816  2403  972  588  729  588  873  828  957  942  699  939  978  894  744  753  972  1308  939  1119  1002  1725  948  633  615  975  612  717  1662  762  1224  693  1029  1284  636  789  618  627  1524  810  813  906  291  267  288  789  906  2397  1446  687  774  858  819  657  2736  765  1080  1965  873  936  717  948  948  918  288  864  843  864  2064  1359  810  693  1347  1299  864  1302  1134  1641  927  513  1149  1674  765  678  2835  2835  3012  690  708  3117  738  1275  507  912  2682  981  795  855  1410  1134  711  543  402  324  1158  657  663  1908  1371  939  249  249  3000  2244  3078 | 252  241  247  280  282  221  223  344  372  336  306  305  344  501  349  299  315  233  371  307  230  343  286  260  327  256  244  263  251  317  280  306  236  267  274  269  262  264  409  873  364  322  346  330  250  374  247  370  258  307  277  292  244  287  385  325  286  265  259  305  271  800  323  195  242  195  290  275  318  313  232  312  325  297  247  250  323  435  312  372  333  574  315  210  204  324  203  238  553  253  407  230  342  427  211  262  205  208  507  269  270  301  96  88  95  262  301  798  481  228  257  285  272  218  911  254  359  654  290  311  238  315  315  305  95  287  280  287  687  452  269  230  448  432  287  433  377  546  308  170  382  557  254  225  944  944  1003  229  235  1038  245  424  168  303  893  326  264  284  469  377  236  180  133  107  385  218  220  635  456  312  82  82  999  747  1025 | -0.677  -0.634  -0.57  -0.647  -0.709  -0.711  -0.663  -0.745  -0.707  -0.545  -0.679  -0.613  -0.786  -0.605  -0.503  -0.697  -0.663  -0.767  -0.697  -0.756  -0.825  -0.726  -0.59  -0.792  -0.534  -0.714  -0.594  -0.849  -0.607  -0.616  -0.527  -0.558  -0.762  -0.961  -0.678  -0.772  -0.612  -0.997  -0.706  -0.446  -0.799  -0.919  -0.301  -0.945  -0.882  -0.595  -0.471  -0.492  -0.79  -0.649  -0.666  -0.741  -0.474  -0.74  -0.586  -0.675  -0.642  -0.638  -0.8  -0.748  -0.815  -0.398  -0.546  -0.805  -0.805  -0.963  -0.746  -0.753  -0.618  -0.657  -0.691  -0.928  -0.691  -0.535  -0.918  -0.714  -0.601  -0.266  -0.71  -0.565  -0.848  -0.289  -0.831  -0.836  -0.815  -0.756  -0.747  -1.118  -0.736  -1.168  -0.687  -0.73  -0.606  -0.747  -0.844  -0.646  -0.691  -0.884  -0.636  -0.636  -0.475  -0.561  -1.016  -0.925  -0.849  -0.588  -0.673  -0.58  -0.65  -1.118  -0.619  -0.762  -0.806  -0.714  -0.629  -0.608  -0.58  -0.654  -0.708  -0.516  -0.574  -0.682  -0.793  -0.643  -0.78  -0.619  -0.639  -0.599  -0.327  -0.217  -0.513  -0.785  -0.912  -0.819  -0.696  -1.009  -0.446  -0.914  -0.504  -0.561  -0.485  -0.813  -0.592  -0.63  -0.535  -0.72  -0.769  -0.788  -0.768  -0.554  -0.992  -0.765  -0.892  -0.781  -0.203  -0.519  -0.629  -0.895  -0.74  -0.795  -0.803  -0.535  -1.011  -0.645  -0.755  -0.769  -0.807  -0.516  -0.817  -0.904  -0.894  -0.913  -0.879  -0.884  -0.866 | nucleus, cytoplasm  nucleus, cytoplasm  nucleus, cytoplasm  nucleus, cytoplasm  nucleus, cytoplasm  nucleus, cytoplasm  nucleus, cytoplasm  nucleus  nucleus  nucleus, peroxisome  nucleus  nucleus  nucleus  nucleus  nucleus, peroxisome  nucleus, cytoplasm  nucleus  nucleus  nucleus, peroxisome  nucleus  nucleus  nucleus  nucleus, cytoplasm  nucleus  nucleus  nucleus  nucleus, peroxisome  nucleus  nucleus  nucleus, peroxisome  nucleus, peroxisome  nucleus, peroxisome  nucleus, peroxisome  nucleus, peroxisome  nucleus  nucleus  nucleus  nucleus  nucleus, peroxisome  nucleus  nucleus, peroxisome  nucleus, peroxisome  nucleus, peroxisome  nucleus  nucleus  nucleus  nucleus  nucleus  nucleus  nucleus  nucleus  nucleus  nucleus, peroxisome  nucleus  nucleus  nucleus  nucleus, peroxisome  nucleus  nucleus  nucleus  nucleus  nucleus  nucleus  nucleus  nucleus  nucleus  nucleus  nucleus  nucleus  nucleus  nucleus  nucleus  nucleus  nucleus, peroxisome  nucleus  nucleus  nucleus  nucleus  nucleus  nucleus  nucleus  nucleus  nucleus  nucleus  nucleus, peroxisome  nucleus  nucleus  nucleus  nucleus  nucleus  nucleus  nucleus  nucleus  nucleus  nucleus  nucleus, peroxisome  nucleus, peroxisome  nucleus  nucleus  nucleus  nucleus, cytoplasm, peroxisome  nucleus  nucleus, cytoplasm, peroxisome  nucleus  nucleus  nucleus  nucleus, peroxisome  nucleus, peroxisome  nucleus  nucleus  nucleus, peroxisome  nucleus  nucleus  nucleus  nucleus  nucleus, peroxisome  nucleus  nucleus  nucleus, peroxisome  nucleus  nucleus  nucleus  nucleus  nucleus, cytoplasm  nucleus  nucleus, peroxisome  nucleus  nucleus  nucleus  nucleus  nucleus, cytoplasm, peroxisome  nucleus  nucleus  nucleus  nucleus  nucleus  nucleus  nucleus  nucleus, peroxisome  nucleus  nucleus  nucleus  nucleus  nucleus, cytoplasm  nucleus  nucleus  nucleus  nucleus, peroxisome  nucleus  nucleus, cytoplasm  nucleus  nucleus, cytoplasm  nucleus  nucleus, cytoplasm  nucleus  nucleus  nucleus, cytoplasm, chloroplasts  nucleus  nucleus  nucleus, cytoplasm, chloroplasts  nucleus  nucleus  nucleus  nucleus  nucleus, cytoplasm, chloroplasts  nucleus, cytoplasm  nucleus, cytoplasm  nucleus  nucleus  nucleus, cytoplasm  nucleus, cytoplasm  nucleus, peroxisome  nucleus, cytoplasm  nucleus  nucleus |
