# Supplementary material for: Genome‑wide analysis of the MYB gene family in pumpkin
Source: PeerJ. 2024 Apr 25;12:e17304. doi: 10.7717/peerj.17304 (PMC11056105; doi:10.7717/peerj.17304)
Supplement: Supplemental Information 4 [file peerj-12-17304-s004.docx]

**Table S2**. The specific primers for the *ComMYB* genes for qRT-PCR analysis.

| **Primer name** | **Sequence (5'to3')** |
| --- | --- |
| CmoMYB99-qF | AGGACCTGTCTCCAACCCAA |
| CmoMYB99-qR | GTTCACATCGCACCCCAATA |
| CmoMYB165-qF | TCTCACGCTATCTCCCTCCG |
| CmoMYB165-qR | CGCCCTTCTTCCAACTCCTT |
| CmoMYB142-qF | CCCAAACATCATCAGGTAAC |
| CmoMYB142-qR | AGCAGACTCATTCAAAACAC |
| CmoMYB154-qF | TTGTTTCAAAATGTCGCCTT |
| CmoMYB154-qR | TTGTTTCTCTCTGTCCTCGC |
| CmoMYB144-qF | CGAACACCGCCCCTTCTCTC |
| CmoMYB144-qR | GGATTTCCGCTGGTCTCTAA |
| CmoMYB116-qF | CTCATTCTCCAACTCCACTC |
| CmoMYB116-qR | CTTGCTTTTGAACTCTCGTC |
| CmoMYB70-qF | GGACAGGGGTGTTGGAGTGA |
| CmoMYB70-qR | TTTGAGGTCGGGTCTAAGGT |
| CmoMYB46-qF | GATTTCAATACCCGCCGCC |
| CmoMYB46-qR | TGCCCCTGTTGTTCTCCTCC |
| CmoMYB64-qF | TGTCATCATTCACCCTGCTG |
| CmoMYB64-qR | TCTCCTTCTTCCTCCCATTT |
| CmoMYB59-qF | AGCCCTCAAGAAGACCAA |
| CmoMYB59-qR | TTCCCGCACCGTAACAGT |
| CmoMYB3-qF | CCGACAGAGAAACGACAT |
| CmoMYB3-qR | GCAAGAAAAGCAAAGACT |
| CmoMYB29-qF | GGAAGAACCGACAATGAGAT |
| CmoMYB29-qR | AGACGACGAGGAAGGAATAC |
| CmoMYB9-qF | AACGGACAATGAAGTAAAGA |
| CmoMYB9-qR | GTAAAAAGATGAACCCAAAA |
| Cmoβ-Actin-qF | GTGCCTGCTATGTATGTTGCC |
| Cmoβ-Actin-qR | GGTCCAAACGGAGAATGGCATG |
