# Supplementary material for: Genome‑wide analysis of the MYB gene family in pumpkin
Source: PeerJ. 2024 Apr 25;12:e17304. doi: 10.7717/peerj.17304 (PMC11056105; doi:10.7717/peerj.17304)
Supplement: Supplemental Information 5 [file peerj-12-17304-s005.docx]

**Table S3**. The homologous *MYB* gene pairs were identified in *C.moschata*

| **NO.** | **Duplicated gene pair** | **NO.** | **Duplicated gene pair** | **NO.** | **Duplicated gene pair** |
| --- | --- | --- | --- | --- | --- |
| 1 | CmoMYB101 - CmoMYB12 | 46 | CmoMYB153 - CmoMYB156 | 91 | CmoMYB63 - CmoMYB35 |
| 2 | CmoMYB104 - CmoMYB10 | 47 | CmoMYB153 - CmoMYB155 | 92 | CmoMYB64 - CmoMYB81 |
| 3 | CmoMYB105 - CmoMYB30 | 48 | CmoMYB154 - CmoMYB157 | 93 | CmoMYB64 - CmoMYB69 |
| 4 | CmoMYB105 - CmoMYB103 | 49 | CmoMYB156 - CmoMYB155 | 94 | CmoMYB64 - CmoMYB67 |
| 5 | CmoMYB107 - CmoMYB119 | 50 | CmoMYB158 - CmoMYB170 | 95 | CmoMYB64 - CmoMYB83 |
| 6 | CmoMYB11 - CmoMYB12 | 51 | CmoMYB161 - CmoMYB170 | 96 | CmoMYB65 - CmoMYB75 |
| 7 | CmoMYB11 - CmoMYB10 | 52 | CmoMYB162 - CmoMYB106 | 97 | CmoMYB65 - CmoMYB159 |
| 8 | CmoMYB111 - CmoMYB162 | 53 | CmoMYB165 - CmoMYB160 | 98 | CmoMYB66 - CmoMYB78 |
| 9 | CmoMYB111 - CmoMYB106 | 54 | CmoMYB168 - CmoMYB169 | 99 | CmoMYB67 - CmoMYB83 |
| 10 | CmoMYB112 - CmoMYB109 | 55 | CmoMYB17 - CmoMYB16 | 100 | CmoMYB68 - CmoMYB76 |
| 11 | CmoMYB114 - CmoMYB130 | 56 | CmoMYB172 - CmoMYB171 | 101 | CmoMYB68 - CmoMYB62 |
| 12 | CmoMYB114 - CmoMYB121 | 57 | CmoMYB18 - CmoMYB24 | 102 | CmoMYB69 - CmoMYB67 |
| 13 | CmoMYB114 - CmoMYB164 | 58 | CmoMYB2 - CmoMYB3 | 103 | CmoMYB69 - CmoMYB83 |
| 14 | CmoMYB116 - CmoMYB111 | 59 | CmoMYB2 - CmoMYB1 | 104 | CmoMYB7 - CmoMYB1 |
| 15 | CmoMYB116 - CmoMYB162 | 60 | CmoMYB22 - CmoMYB48 | 105 | CmoMYB70 - CmoMYB79 |
| 16 | CmoMYB116 - CmoMYB106 | 61 | CmoMYB22 - CmoMYB25 | 106 | CmoMYB71 - CmoMYB93 |
| 17 | CmoMYB117 - CmoMYB86 | 62 | CmoMYB23 - CmoMYB20 | 107 | CmoMYB72 - CmoMYB60 |
| 18 | CmoMYB118 - CmoMYB47 | 63 | CmoMYB29 - CmoMYB27 | 108 | CmoMYB73 - CmoMYB79 |
| 19 | CmoMYB122 - CmoMYB123 | 64 | CmoMYB30 - CmoMYB103 | 109 | CmoMYB74 - CmoMYB79 |
| 20 | CmoMYB125 - CmoMYB113 | 65 | CmoMYB34 - CmoMYB21 | 110 | CmoMYB74 - CmoMYB80 |
| 21 | CmoMYB126 - CmoMYB129 | 66 | CmoMYB36 - CmoMYB103 | 111 | CmoMYB75 - CmoMYB159 |
| 22 | CmoMYB126 - CmoMYB139 | 67 | CmoMYB38 - CmoMYB36 | 112 | CmoMYB76 - CmoMYB62 |
| 23 | CmoMYB126 - CmoMYB131 | 68 | CmoMYB40 - CmoMYB30 | 113 | CmoMYB79 - CmoMYB80 |
| 24 | CmoMYB129 - CmoMYB139 | 69 | CmoMYB40 - CmoMYB103 | 114 | CmoMYB8 - CmoMYB9 |
| 25 | CmoMYB135 - CmoMYB128 | 70 | CmoMYB41 - CmoMYB22 | 115 | CmoMYB8 - CmoMYB11 |
| 26 | CmoMYB136 - CmoMYB151 | 71 | CmoMYB41 - CmoMYB48 | 116 | CmoMYB8 - CmoMYB12 |
| 27 | CmoMYB136 - CmoMYB133 | 72 | CmoMYB41 - CmoMYB25 | 117 | CmoMYB8 - CmoMYB10 |
| 28 | CmoMYB136 - CmoMYB134 | 73 | CmoMYB42 - CmoMYB44 | 118 | CmoMYB81 - CmoMYB67 |
| 29 | CmoMYB139 - CmoMYB131 | 74 | CmoMYB45 - CmoMYB58 | 119 | CmoMYB81 - CmoMYB83 |
| 30 | CmoMYB139 - CmoMYB132 | 75 | CmoMYB45 - CmoMYB49 | 120 | CmoMYB82 - CmoMYB64 |
| 31 | CmoMYB139 - CmoMYB128 | 76 | CmoMYB45 - CmoMYB47 | 121 | CmoMYB82 - CmoMYB81 |
| 32 | CmoMYB14 - CmoMYB13 | 77 | CmoMYB46 - CmoMYB120 | 122 | CmoMYB82 - CmoMYB69 |
| 33 | CmoMYB140 - CmoMYB132 | 78 | CmoMYB48 - CmoMYB25 | 123 | CmoMYB82 - CmoMYB67 |
| 34 | CmoMYB141 - CmoMYB137 | 79 | CmoMYB49 - CmoMYB47 | 124 | CmoMYB87 - CmoMYB84 |
| 35 | CmoMYB142 - CmoMYB138 | 80 | CmoMYB5 - CmoMYB4 | 125 | CmoMYB89 - CmoMYB115 |
| 36 | CmoMYB144 - CmoMYB148 | 81 | CmoMYB50 - CmoMYB45 | 126 | CmoMYB9 - CmoMYB11 |
| 37 | CmoMYB144 - CmoMYB143 | 82 | CmoMYB50 - CmoMYB58 | 127 | CmoMYB9 - CmoMYB12 |
| 38 | CmoMYB145 - CmoMYB146 | 83 | CmoMYB50 - CmoMYB49 | 128 | CmoMYB9 - CmoMYB10 |
| 39 | CmoMYB145 - CmoMYB150 | 84 | CmoMYB50- CmoMYB47 | 129 | CmoMYB90 - CmoMYB88 |
| 40 | CmoMYB145 - CmoMYB147 | 85 | CmoMYB54 - CmoMYB76 | 130 | CmoMYB91 - CmoMYB94 |
| 41 | CmoMYB146 - CmoMYB150 | 86 | CmoMYB54 - CmoMYB72 | 131 | CmoMYB95 - CmoMYB97 |
| 42 | CmoMYB146 - CmoMYB147 | 87 | CmoMYB55 - CmoMYB31 | 132 | CmoMYB95 - CmoMYB87 |
| 43 | CmoMYB148 - CmoMYB143 | 88 | CmoMYB57 - CmoMYB51 | 133 | CmoMYB95 - CmoMYB84 |
| 44 | CmoMYB150 - CmoMYB147 | 89 | CmoMYB58 - CmoMYB49 | 134 | CmoMYB97 - CmoMYB87 |
| 45 | CmoMYB151 - CmoMYB133 | 90 | CmoMYB61 - CmoMYB102 | 135 | CmoMYB97 - CmoMYB84 |
