# Supplementary figures and images for: Genome‑wide analysis of the MYB gene family in pumpkin
Source: PeerJ. 2024 Apr 25;12:e17304. doi: 10.7717/peerj.17304 (PMC11056105; doi:10.7717/peerj.17304)

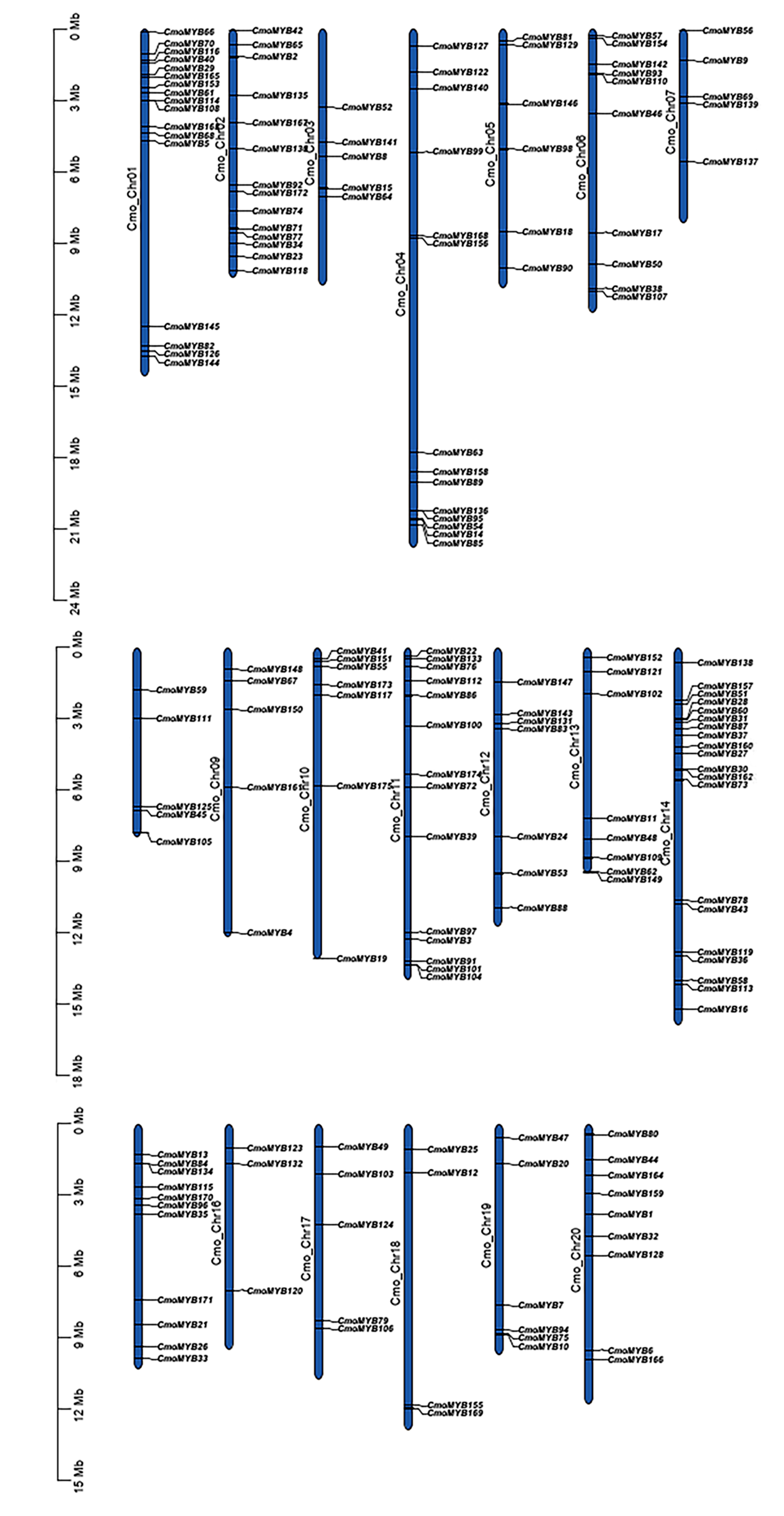

Supplement: Supplemental Information 6 — The chromosomal position of each CmoMYB was mapped according to the C.moschata genome. Gff3 files was downloaded from the cucurbitaceae database, TBtools software was used to visualize the result. The numbers of chromosomes are indicated in the left middle of each chromosome, the right side of the chromosome is the location of the gene. Scale bar is in million bases (Mb). [file peerj-12-17304-s006.png]

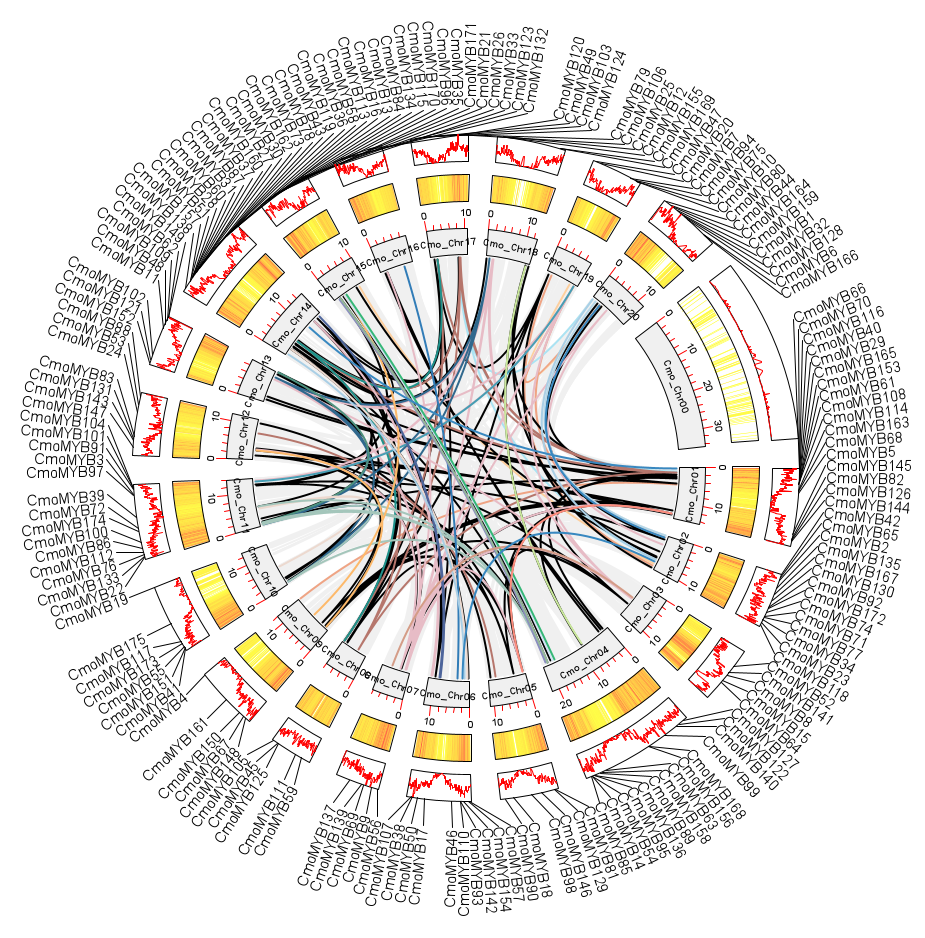

Supplement: Supplemental Information 7 — The chromosomal position of each CmoMYB was mapped according to the C.moschata genome. The different colorful lines in circle indicate a collinearity relationship among genes. [file peerj-12-17304-s007.png]

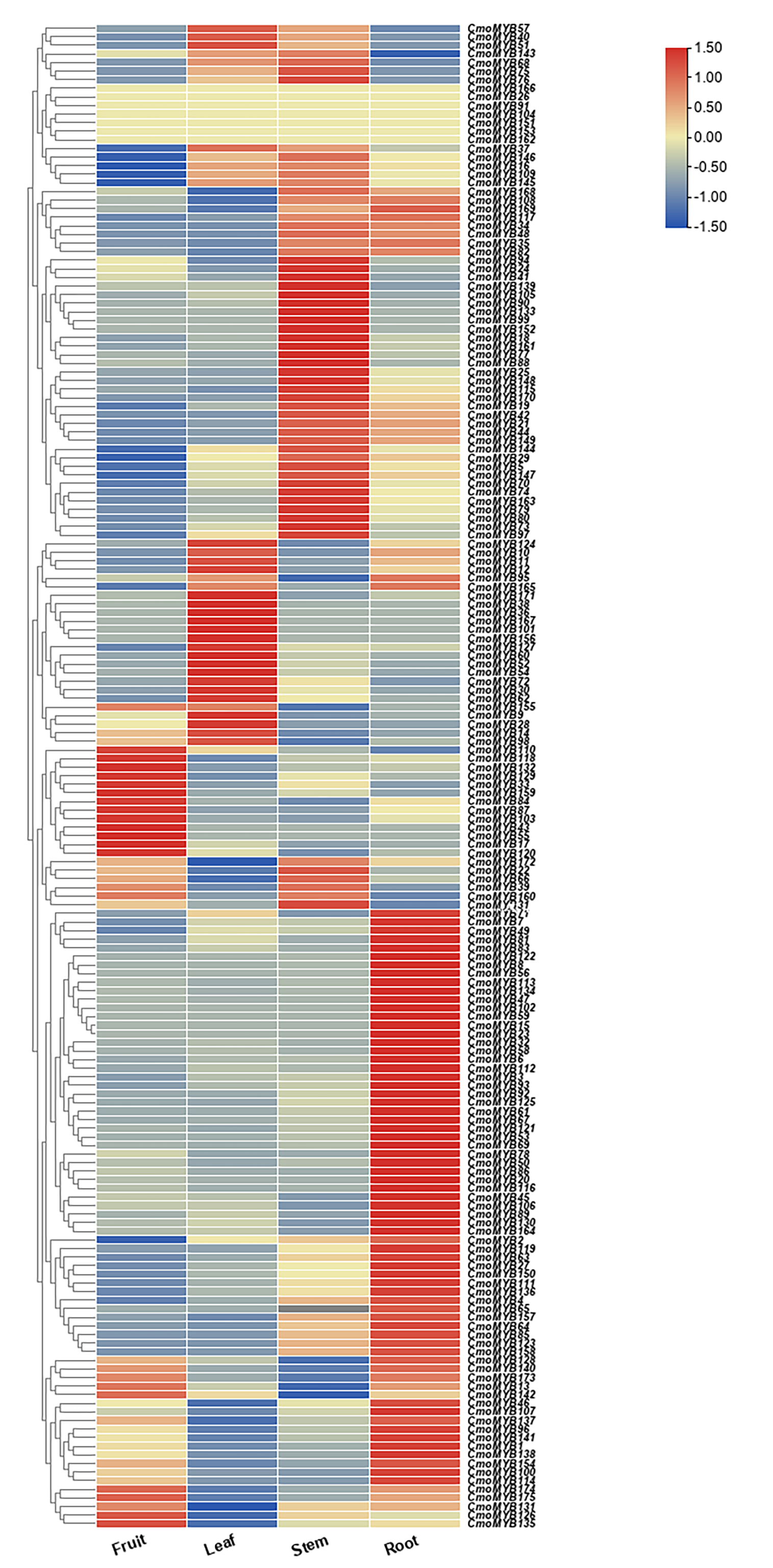

Supplement: Supplemental Information 8 — Higher and lower levels of transcript accumulation are indicated by red and blue, respectively. The expression data were obtained from the cucurbitaceae database, and the heat map for specific expression analysis was constructed by TBtools software. [file peerj-12-17304-s008.png]

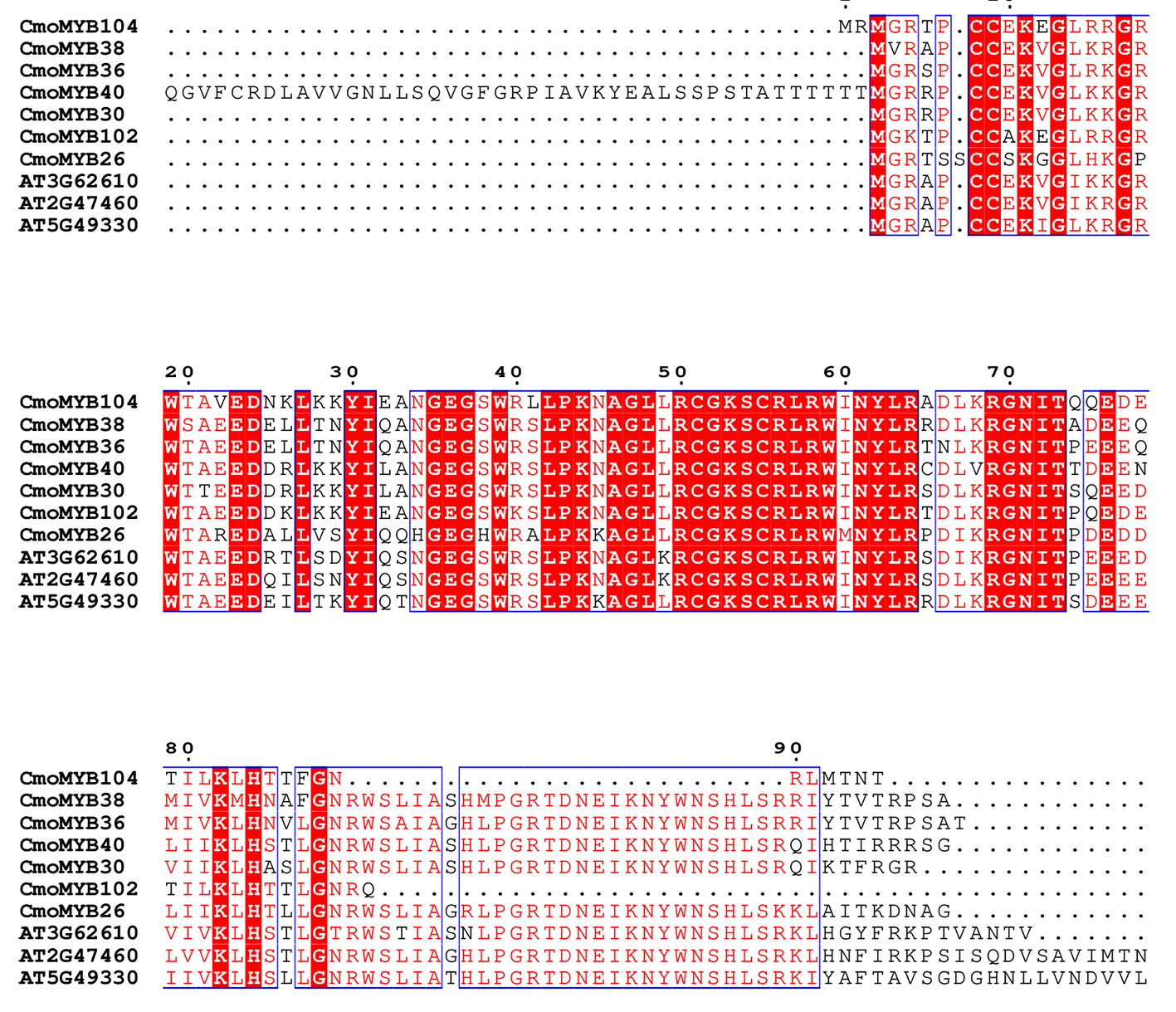

Supplement: Supplemental Information 9 — Multiple sequence comparison of the amino acid sequences of CmoMYB104, CmoMYB38, CmoMYB36, CmoMYB40, CmoMYB30, CmoMYB102, CmoMYB26, AT3G62610, AT2G47460, and AT5G49330. Firstly, the MEGA software was used to do multiple sequence comparison of amino acid sequences. Then, visualization using BEG (BEGinner) mode in ESPript 3.0 online website (https://espript.ibcp.fr/ESPript/cgi-bin/ESPript.cgi) [file peerj-12-17304-s009.png]

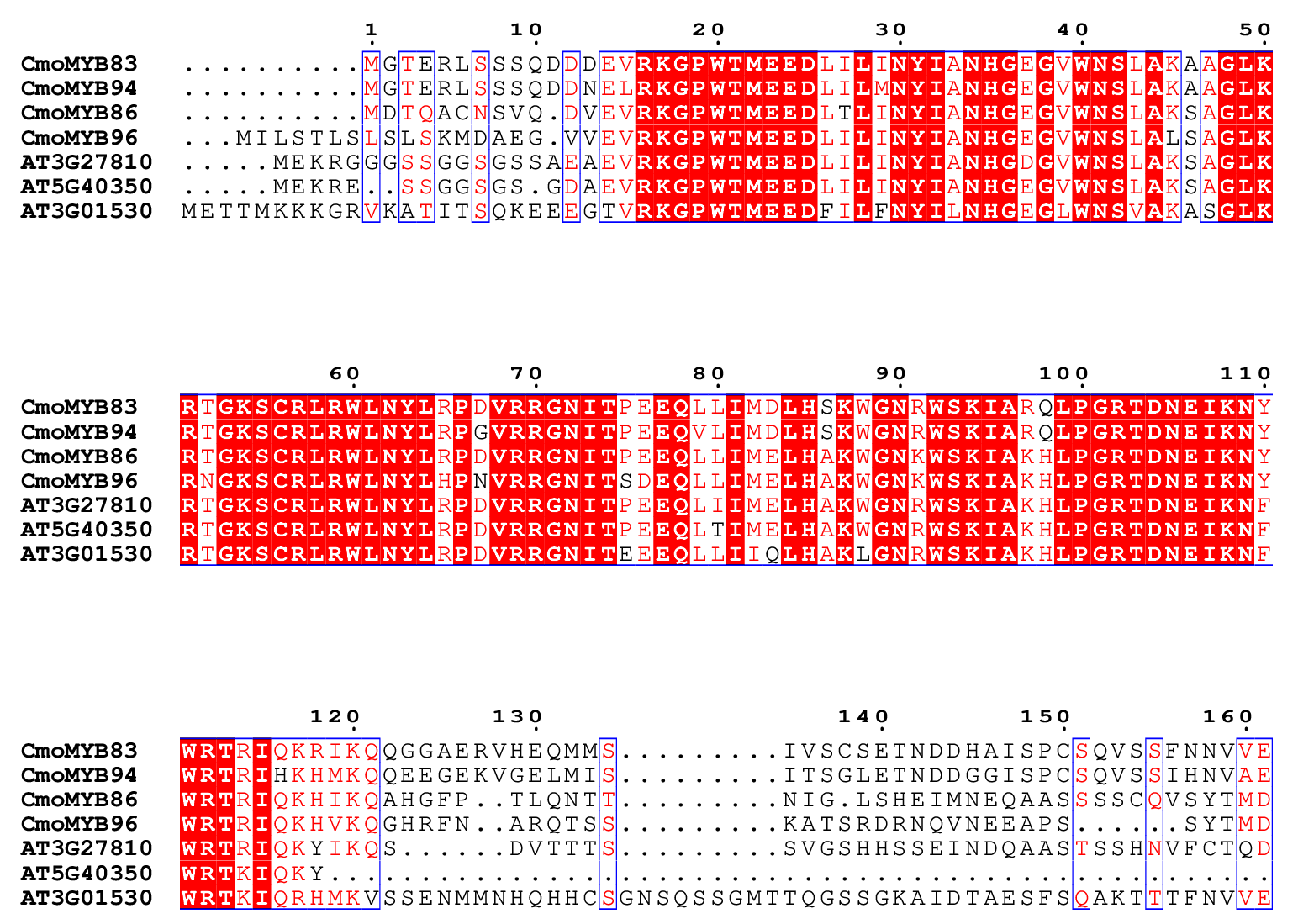

Supplement: Supplemental Information 10 — Multiple sequence comparison of the amino acid sequences of CmoMYB83, CmoMYB94, CmoMYB86, CmoMYB96, AT3G27810, AT5G40350, and AT3G01530. Firstly, the MEGA software was used to do multiple sequence comparison of amino acid sequences. Then, visualization using BEG (BEGinner) mode in ESPript 3.0 online website (https://espript.ibcp.fr/ESPript/cgi-bin/ESPript.cgi) [file peerj-12-17304-s010.png]
